# Supplementary material for: The interactome of intact mitochondria by cross-linking mass spectrometry provides evidence for coexisting respiratory supercomplexes
Source: Mol Cell Proteomics. 2017 Dec 8;17(2):216–32. doi: 10.1074/mcp.RA117.000470 (PMC5795388; doi:10.1074/mcp.RA117.000470)
Supplement: Supplemental Data [file supp_RA117.000470_133922_0_supp_23978_fzffwf.zip › spectra_annotation/mito_spectra_annotation/392-1-12-1-12-1.pdf]

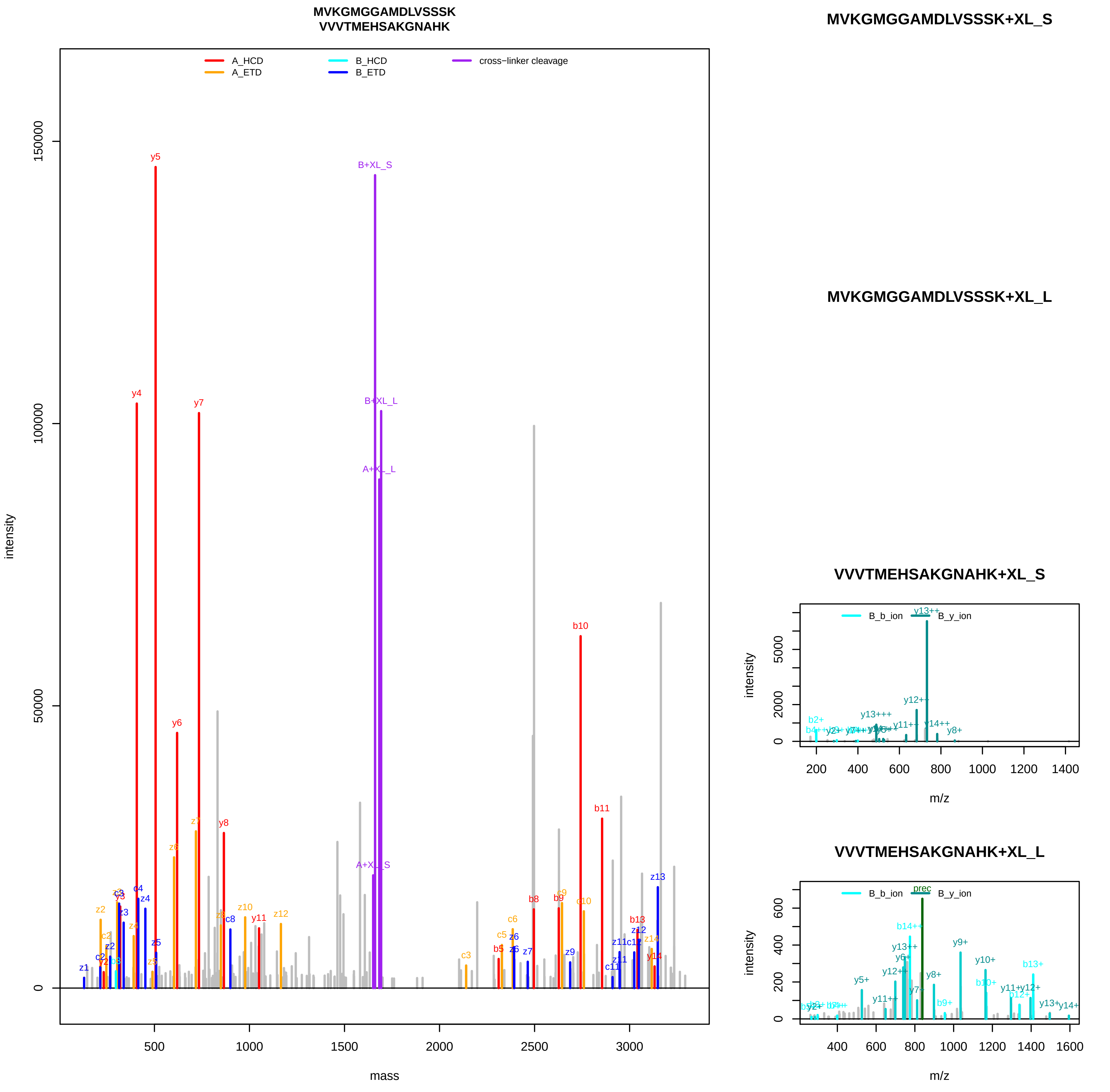
MVKGMGGAMDLVSSSK
VVVTMEHSAKGNAHK
A_HCD
A_ETD
B_HCD
B_ETD
cross−linker cleavage
intensity
mass
0
50000
100000
150000
500
1000
1500
2000
2500
3000
y5
B+XL_S
y4
y7
B+XL_L
A+XL_L
A+XL_S
b10
b11
y6
y8
z7
z6
b8
b9
c9
c10
z10
y11
z12
z13
b13
z12
z14
y14
MVKGMGGAMDLVSSSK+XL_S
MVKGMGGAMDLVSSSK+XL_L
VVVTMEHSAKGNAHK+XL_S
B_b_ion
B_y_ion
y13++
y12++
y13+++
y11++
y14++
y8+
b2+
m/z
intensity
0
2000
5000
200
400
600
800
1000
1200
1400
VVVTMEHSAKGNAHK+XL_L
B_b_ion
B_y_ion
prec
b14++
y13++
y9+
y10+
b13+
y5+
y12++
y8+
b10+
y11+y12+
y11++
b9+
b12+
y13+
y14+
m/z
intensity
0
200
400
600
400
600
800
1000
1200
1400
1600
